# Causal effects of hypertension on risk of erectile dysfunction: A two-sample Mendelian randomization study

**DOI:** 10.3389/fcvm.2023.1121340

**Published:** 2023-03-21

**Authors:** Zheng Wang, Yunyun Wang, Jiachao Xiong, Xinxin Gan, Yewei Bao, Aimin Jiang, Ye Zhou, Zhao Huangfu, Yiren Yang, Zhiyong Liu, Demeng Xia, Linhui Wang

**Affiliations:** ^1^ Department of Urology, Changhai Hospital, Naval Medical University, Shanghai, China; ^2^ Department of Critical Care Medicine, Huashan Hospital, Fudan University, Shanghai, China; ^3^ Department of Plastic and Reconstructive Surgery, Shanghai East Hospital, School of Medicine, Tongji University, Shanghai, China; ^4^ Luodian Clinical Drug Research Center, Shanghai Baoshan Luodian Hospital, Shanghai University, Shanghai, China

**Keywords:** antihypertensive drug, hypertension, erectile dysfunction, Mendelian randomization, PDE5 inhibitor

## Abstract

**Background:**

Erection dysfunction has been associated with hypertension in several epidemiological and observational studies. But the causal association between hypertension and erectile dysfunction requires further investigation.

**Methods:**

A two-sample Mendelian randomization (MR) was conducted to analyze the causal effect of hypertension on risk of erection dysfunction. Large-scale publicly available genome-wide association study data were used to estimate the putative causality between hypertension and risk of erectile dysfunction. A total of 67 independent single nucleotide polymorphisms were selected as instrumental variables. Inverse-variant weighted, maximum likelihood, weighted median, penalized weighted median, and MR-PRESSO approaches were utilized in MR analyses. Heterogeneity test, horizontal pleiotropy test, and leave-one-out method were used to prove the stability of the results.

**Results:**

In total, all *P* values were less than 0.05, demonstrating a positive causal link between hypertension and risk of erectile dysfunction in multiple MR methods, such as inverse-variant weighted (random and fixed effect) (OR 3.8315, 95% CI 2.3004–6.3817, *P *= 0.0085), maximum likelihood (OR 3.8877, 95% CI 2.3224–6.5081, *P *= 0.0085), weighted median (OR 4.9720, 95% CI 2.3645–10.4550, *P *= 0.0309), penalized weighted median (OR 4.9760, 95% CI 2.3201–10.6721, *P *= 0.0355), and MR-PRESSO (OR 3.6185, 95% CI 2.2387–5.8488, *P *= 0.0092). Sensitivity analysis detected no evidence of heterogeneity, pleiotropy, or outlier single nucleotide polymorphisms.

**Conclusion:**

The study revealed a positive causal link between the presence of hypertension and the risk of erectile dysfunction. More attention should be paid during the management of hypertension with the purpose of preventing erectile dysfunction or improving erectile function.

## Introduction

Erectile dysfunction (ED) is defined as the inability of attaining or maintaining sufficient and satisfying erection for sexual intercourse (1). Further diagnosis and precise grading are mainly based on the International Index for Erectile Function (IIEF) (2, 3) or Brief Male Sexual Function Inventory for Urology (BMSFI) (4). Sexual function is one of the most fundamental biological characteristics of *Homo sapiens*, and erectile function holds great importance for a majority of males. ED is regarded as a major health problem that affects up to millions of men in the world and keeps causing a negative impact on the quality of life (QoL) of the patients and their partners. In the Massachusetts Male Aging Study (MMAS), a population-based prospected randomized study on aging men, it was found that over 52% men between 40 and 70 years old reported ED. The incidence rate of ED was about 26 cases per 1,000 man-years (5). Among men under 49 years old, the prevalence of ED was approximately 6%. By the age of 50–59, 16% of men were estimated to suffer from ED, and this number grew to 32% for those between 60 and 69, and even higher to 44% for those aged 70–79 (6). The epidemiology data of ED mainly depend on query or self-reporting. Hence, real-life incidence may be even higher than the estimated number. According to predictions, by 2025, the estimated cases of ED would reach 322 million worldwide (7). The continuous rising prevalence of ED brings a heavy burden on healthcare, finance, and society. Hence, the prevention of, and early intervention for, the risk factors of ED hold considerable significance and a sense of urgency. A variety of risk factors of ED has been investigated in previous studies, such as hypertension (8), cardiovascular diseases (9), diabetes mellitus (10), obesity (11), unhealthy lifestyle (12), and so on.

Hypertension is one of the risk factors for ED and affects the blood supply to the penis, with a strong association due to shared intrinsic mechanisms such as endothelial dysfunction (13). The mechanisms linking hypertension and ED involve intertwined procontractile signaling pathways, resulting in reduced vascular flexibility (14, 15). Hypertension is estimated to affect up to 1.39 billion people globally (16, 17). The number of people with both hypertension and ED is significantly high, and the health management and quality of life of these patients is extremely important. A lot of clinical studies, including large-scale cross-national cohorts, have explored the correlation between hypertension and ED (18–23). However, some studies have reached contradictory conclusions (24–26). Most of these studies mentioned above mainly focused on the potential of ED as a predictor for cardiovascular diseases, especially the coronary artery disease, instead of further investigating the relationship between hypertension and ED. Some studies proposed that not hypertension but antihypertensive drugs trigger ED (27, 28). Only if the causal relationship and the direction of this relationship are definitely established will answers be found to the above questions.

To provide high-level clinical evidence and validate causality, Mendelian randomization (MR) was adopted to investigate the causality between hypertension and ED. MR could compensate for the deficiency of observational studies and obtain unbiased estimates without conducting a randomized controlled trial (RCT) (29, 30). Although RCTs are considered to have the highest level of evidence, with the increase in the study scale, comes the complexity of organizational and financial support, as well as the challenges of passing various legal and ethical reviews. These factors can all contribute to a higher implementation cost for RCTs. Therefore, despite RCTs providing a higher level of evidence, they also bring more challenges. In contrast, MR studies tend to be less costly and easier to conduct compared with RCTs (31). In addition, MR has the advantage of being able to establish causality between exposure and outcome, while RCTs can establish only association. This is because MR studies use genetic variation as an instrument for exposure, which eliminates the effects of reverse causality and confounding, making the causal inference more robust (32).

The Mendelian randomization method utilizes genetic variants to determine the causality between exposure and outcome (33). The genetic variants are significantly linked with the exposure, independent of confounding factors associated with exposure and outcome, and affect the outcome only *via* exposure. MR typically uses single nucleotide polymorphism (SNP) as a proxy for the exposure factor. SNP is a variation at a single nucleotide site in the genome and is randomly allocated among individuals, so that they are not affected by environmental or lifestyle factors. Moreover, SNPs do not change over a lifetime, making them useful as long-term biological markers (34). To gain access to genetic data, publicly available genome-wide association studies (GWAS) were explored. The statistics was then analyzed by MR. By excluding confounding bias factors, confounding and reverse causality could be avoided in order to gain an unaffected causal link between exposure and outcome (35). In the present study, a two-sample MR based on the large-scale publicly available GWAS data was applied to estimate the putative causality between hypertension and risk of ED.

## Materials and methods

The study was conducted to obtain a comprehensive and reliable conclusion of the casual link between hypertension and risk of ED.

### Data sets

We used the GWAS statistics obtained from large-scale genetic consortia of ED patients. Bovijn et al. identified the risk locus for ED by performing a genome-wide study among 6,175 European ED patients (36). Hypertension GWAS summary data were obtained from the Medical Research Council Integrative Epidemiology Unit at the University of Bristol (MRC IEU). As many as 54,358 European patients diagnosed with hypertension and 40,8652 controls were included in the database. All the above-mentioned data sets are available on https://gwas.mrcieu.ac.uk/ and used for MR analysis.

SNPs with a strong association with ED of genome-wide significance (*P* < 5 × 10^−8^) were selected and SNPs were pruned in linkage disequilibrium (*R*^2^ > 0.001 within a 10,000 kb window) with the clump data function in the TwoSampleMR software package in R. The filtered SNPs were finally qualified for the following MR analysis. The strength of the genetic instruments was indicated by the F-statistic.

### Mendelian randomization

**Figure 1:**
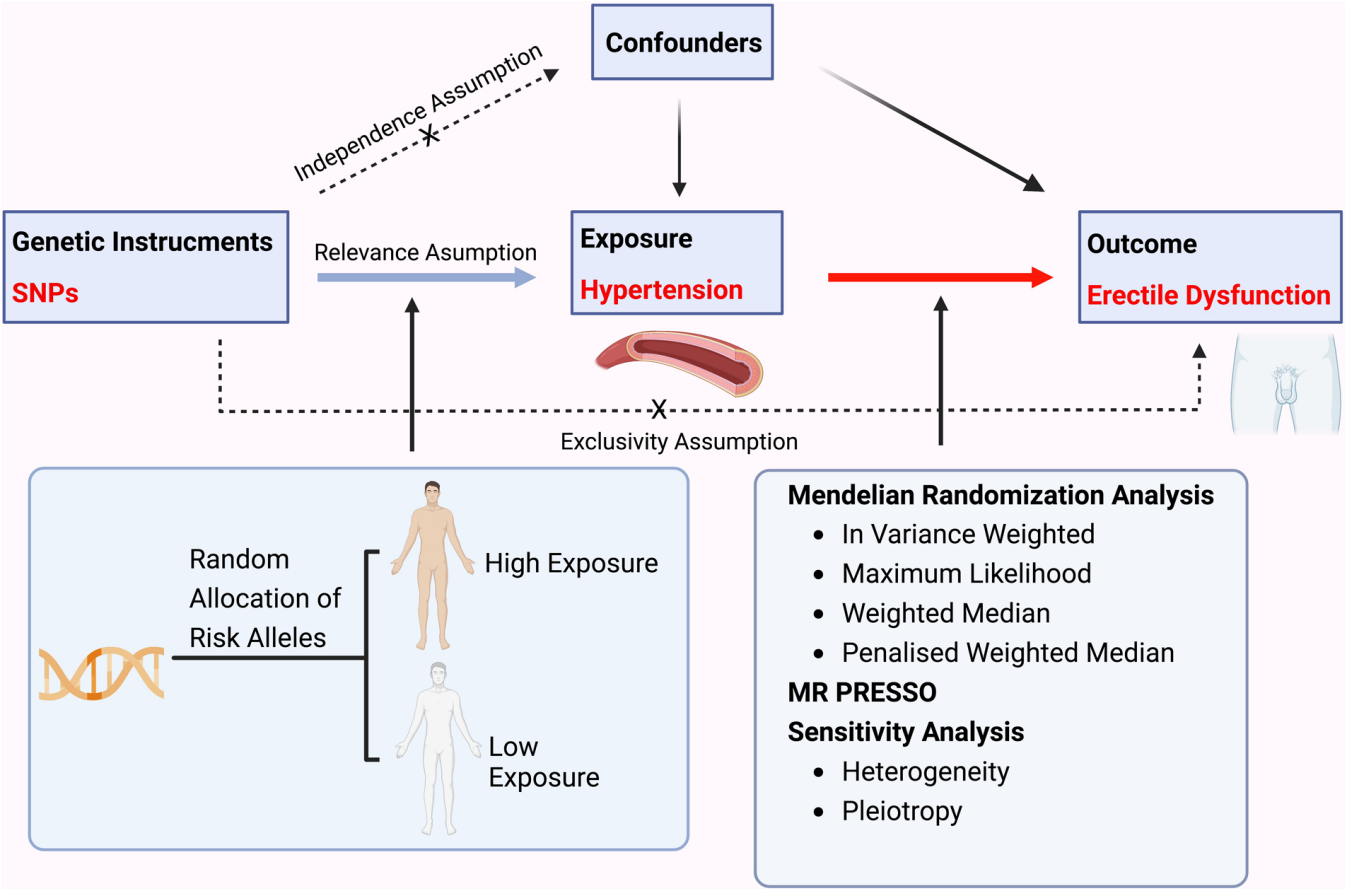
A flow chart illustrating the causality of the relationship between hypertension and erectile dysfunction.

Mendelian randomization analysis aims to apply genetic variants as instrumental variables to estimate the causal effect between exposure and outcome, namely hypertension and ED in this study (Figure 1).

An inversed-variance weighted (IVW) (random and fixed effect) meta-analysis of each Wald ratio was performed to obtain MR estimate since there is no evidence of directional pleiotropy (37). The outlier variables were detected by the MR-PRESSO method in IVW analysis by comparing the actual distance from the regression, assuming the absence of horizontal pleiotropy, and evaluating the causal estimates after removing outliers (38). In addition, several statistical analyses were performed to assess the stability of the MR results, including maximum likelihood, weighted median, and penalized weighted median approaches.

### Pleiotropy and sensitivity analysis

Heterogeneity was detected by inverse-variance weighted and MR-Egger regression and quantified by using Cochran's Q test. A *P*-value <0.05 would be considered as significant heterogeneity. The potential horizontal pleiotropic effects of the instrumental variables were assessed by the intercept term in MR-Egger regression. If the intercept term converged to 0 (<0.1) as well as *P *> 0.05, it was indicated that no evidence of horizontal pleiotropy was detected in the analysis and the results of the MR analyses were reliable.

Moreover, a “leave-one-out” sensitivity analysis was repeated to test the potentially influential SNPs. In this analysis, each SNP would be left out in turn and the MR would be repeated over again.

Mendelian randomization analyses were performed by using the “TWO-SampleMR” package and “MR-PRESSO” in R software 4.0.5. All *P* values were two-sided.

## Results

### Selection of variables

In total, 67 qualified SNPs related to ED and hypertension were collected in this study. All the instrumental variables met the generally accepted genome-wide significance threshold (*P *< 5 × 10^−8^, r^2 ^< 0.001, kb = 10,000) for exposure. The F-statistic indicated no weak instrument bias (all F-statistic > 10).

### Mendelian randomization estimates

**Figure 2:**
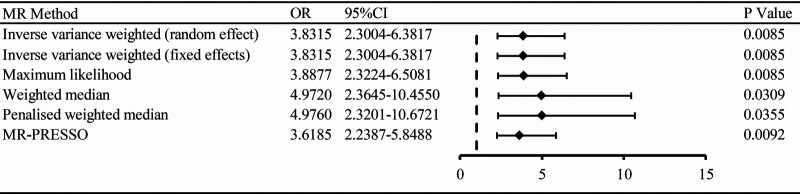
The association of hypertension with ED outcomes by MR analysis through different methods (random effect and fixed effects inverse variance weighted method, maximum likelihood, weighted median, penalized weighted median, MR-PRESSO). OR: odds ratio; CI: confidence interval.

**Figure 3:**
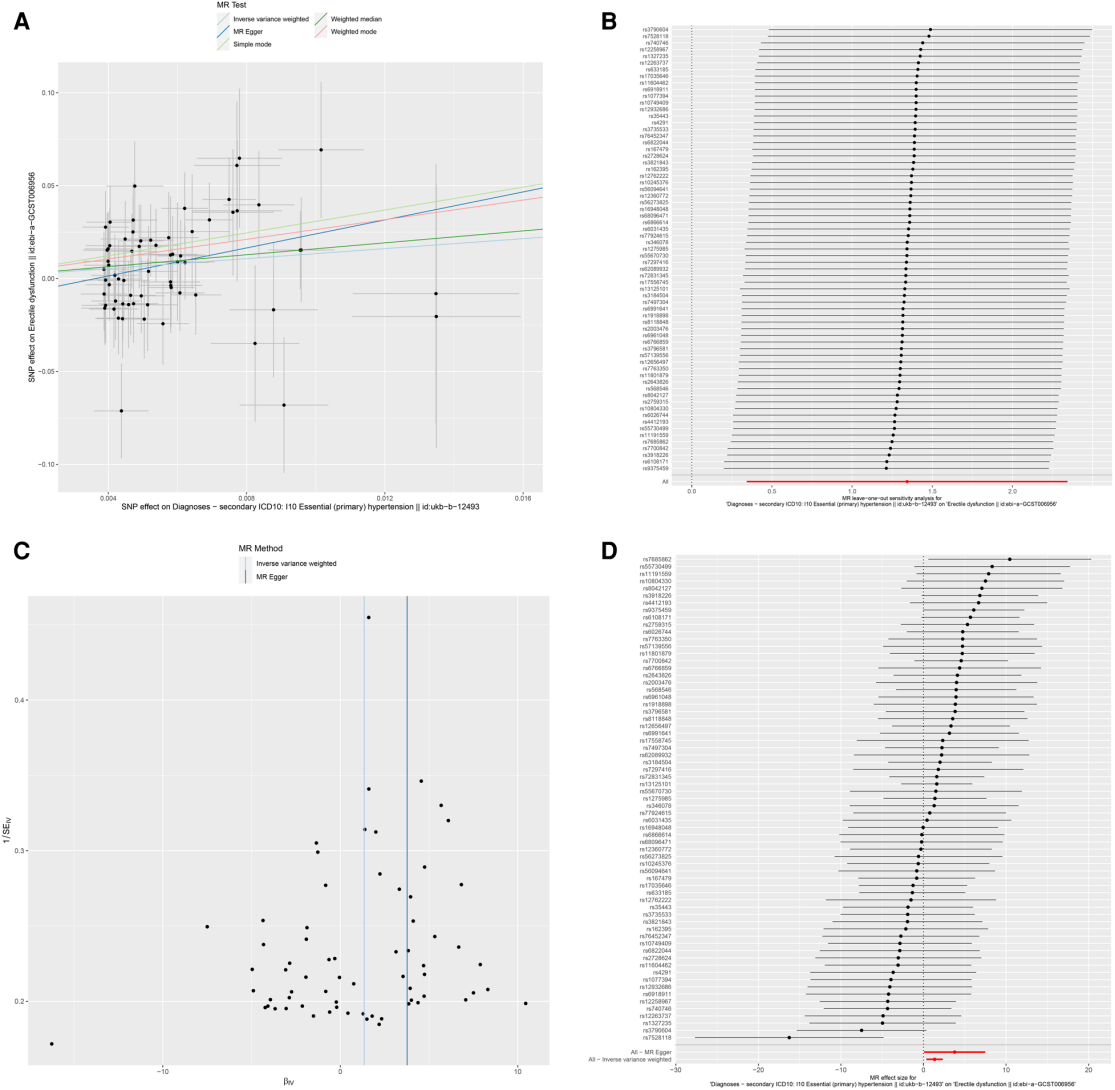
Association between hypertension and risk of erectile dysfunction. (A) multiple MR tests showed the SNP effects; (B) leave-one-out sensitivity analysis; (C) funnel plot for hypertension risk of ED; (D) effect size of each SNP. MR Mendelian Randomization, SNP single nucleotide polymorphism, ED erectile dysfunction.

Mendelian randomization analysis demonstrated a significant association of hypertension with ED outcomes. The results were consistent with IVW (random and fixed effect) (OR 3.8315, 95% CI 2.3004–6.3817, *P* = 0.0085), maximum likelihood (OR 3.8877, 95% CI 2.3224–6.5081, *P* = 0.0085), weighted median (OR 4.9720, 95% CI 2.3645–10.4550, *P* = 0.0309), penalized weighted median (OR 4.9760, 95% CI 2.3201–10.6721, *P* = 0.0355), and MR-PRESSO (OR 3.6185, 95% CI 2.2387–5.8488, *P* = 0.0092) (Figures 2, 3A).

### Pleiotropy and sensitivity analysis

To explore the sensitivity of the analysis, we conducted the Cochren's Q test, which indicated no evidence of heterogeneity. The horizontal pleiotropy between SNPs and outcome was assessed by MR-Egger regression. No evidence of horizontal pleiotropy was found. The results of the leave-one-out analysis validated that no potentially influential SNP biased the casual link, and our conclusion was stable (Figure 3B). The funnel demonstrated the displayed symmetric pattern of each SNP on ED and indicated no apparent horizontal pleiotropy (Figure 3C). The effect size of each SNP is shown in Figure 3D.

## Discussion

In this study, a two-sample MR analysis was performed to investigate the potential causal effect of hypertension on ED outcomes and revealed the suggested association of hypertension with an increased risk of ED. By employing several Mendelian tools, the results were proved reliable for achieving stability in the sensitivity analysis. In previous MR studies, the casual effects of insomnia (39), snoring (40), educational attainment (41), and COVID-19 (42) on risk of ED have been sufficiently investigated.

Based on the etiology, ED could be categorized as organic, psychogenic, or mixed ED. Noteworthily, the vast majority of patients are actually affected by mixed causes. In other words, organic lesion could be found in most patients. The pathological classification of ED includes vasculogenic, endocrinologic, neurogenic, anatomical, drug-related, psychogenic, or mixed causes (43). The arterial insufficiency is the primary cause of ED. The blood supply for penis mainly comes from the iliac and the pudendal artery and flows to the penile arterial system. An impairment in any segment of this arterial system may lead to ED. Hypertension as a major detrimental factor for vascular impairment could largely damage the blood flow to the penis (44).

During the past several years, a few large-scale observational studies on the incidence of ED among hypertensive patients have revealed that hypertension is closely correlated to an elevated risk of ED (18–20). A multicenter, prospective, open, observational study in Spain found a high incidence of ED in male patients with hypertension (975/2,130, 45.8%) compared with that in a normotensive male population (45). Furthermore, ED was found to be more prevalent in patients with long-duration or severe hypertension, which further illustrates the link between hypertension and risk of ED (46). The results of these mentioned studies were consistent with our findings.

Actually, ED shares not only various common risk factors (unhealthy lifestyle, obesity, aging, alcohol and tobacco use, etc.) (47), but also multiple intrinsic mechanisms such as endothelial and vascular smooth muscle dysfunction. A variety of vasoconstrictors (angiotensin II, endothelin 1, aldosterone, etc.) and vasodilators (nitric oxide, hydrogen sulfide, Nrf2, etc.) are strongly associated with the pathophysiologic pathways of hypertension and ED.

As a modifiable risk factor, the management of hypertension also significantly interferes with the treatment of ED. According to the recommendations given by the Princeton Consensus Conferences on optimizing sexual dysfunction and preserving cardiovascular health, ED patients with asymptomatic-controlled hypertension can receive treatment for ED in the first place and continue sexual activity without the fear of significant cardiac risk. Otherwise, ED patients with uncontrolled hypertension are stratified as a high-risk group. For these patients, treatment for ED should always be secondary to the management of hypertension or other cardiovascular diseases. More importantly, any form of sexual activity is strictly forbidden (48).

Noteworthily, although satisfying blood pressure control levels are closely related to erectile function benefits, antihypertensive therapy may independently trigger or worsen ED. According to the results of the MMAS study, one of the most valuable epidemiologic studies on ED, receiving treatment for hypertension strongly elevates the risk of ED (5). Different antihypertensive drugs have distinct effects on ED (49). Some *β*-blockers could negatively influence erectile function by blocking *β*-2 receptors, thus resulting in the constriction of the penile arteries (50), while nebivolol as a new-generation selective *β*-BLOCKER has a positive effect on erectile function (51). Diuretics is also considered to exert detrimental effects on ED as a common side effect (52). Angiotensin receptor blockers (ARBs) and angiotensin-converting enzyme inhibitors (ACEIs) could be beneficial, or at least neutral, to erectile function and sexual activity (53).

In our study, a causal link between hypertension and risk of ED has been established with all confounding factors being excluded. Arterial hypertension brings more burden than any other diseases globally, which affects more than 1 billion people worldwide (17, 54). Considering the higher prevalence of ED in hypertensive patients, antihypertensive therapy regardless of side effects on ED no doubt would impair the QoL, cause a heavy mental burden, affect medication compliance, and eventually aggravate the vicious circle of hypertension and ED in a large number of patients and their partners. Hence, antihypertensive therapy, which is beneficial for erectile function, should be attempted on a large scale on untreated patients. For those patients who receive antihypertensive drugs with detrimental effects on erectile function, a switch in the therapeutic regimen with caution may be a wise decision. The coadministration of selective phosphodiesterase type 5 inhibitors (PDE5I) could be an alternative for avoiding the increased risk brought by the change to antihypertensive therapy. Currently, four potent PDE5I drugs are approved for ED treatment, namely, sildenafil, tadalafil, vardenafil, and avanafil. PDE5Is were effective in approximately 60%–70% of hypertensive patients, and some RCTs validated the safety of PD5EI in these patients (27, 55, 56). However, since hypertension is a modifiable risk factor of ED, priority should always be to prevent ED from initiating.

The main strength of this study is the merit of the MR design, which could evaluate the independent causal effects of hypertension on ED. To the best of our knowledge, this is the very first MR study on hypertension and ED based on large-scale consortium data. The application of MR avoids several limitations in retrospective studies. In addition, 67 qualified SNPs in the European population were used as instrumental variables, which constructed a well-powered MR analysis. Hence, the results were unlikely impacted by population stratification.

Inevitably, certain limitations exist in our study. First, due to the lack of original data in the GWAS data set, we could not fully evaluate the severity of ED and hypertension. Second, the most adaptable inquiry for the epidemiological data of ED is self-reporting and questionnaire survey. Owing to patients' reluctance to disclose their sexual dysfunction problems, the prevalence of ED in hypertensive patients may be underestimated. Third, our study was mainly based on the genetic data of European ancestry, and the results may be inconsistent in other ethnic populations. Also, each MR method has its own strength and weakness, and we cannot completely rule out potential bias.

Our results suggested that hypertension would increase the risk of ED outcomes. But a definite causal relationship requires conducting more RCTs with high quality and more in-depth studies in the future.

Our results confirmed a positive causal link between the presence of hypertension and the risk of ED in the general population. This MR study could serve as high-level clinical evidence of the impact of hypertension on erectile function, revealing causality and providing reference for clinical diagnosis and treatment, with the aim of improving the treatment effect of ED in the hypertension population, as well as providing certain guidance for the medication regimen of antihypertensive drugs to protect erectile function.

## Data Availability

Publicly available data sets were analyzed in this study. These data sets can be found here: https://gwas.mrcieu.ac.uk/. Please refer to the supplementary materials for the original code of this study.
